# The Sensilla-Specific Expression and Subcellular Localization of SNMP1 and SNMP2 Reveal Novel Insights into Their Roles in the Antenna of the Desert Locust *Schistocerca gregaria*

**DOI:** 10.3390/insects13070579

**Published:** 2022-06-25

**Authors:** Sina Cassau, Doreen Sander, Thomas Karcher, Michael Laue, Gerd Hause, Heinz Breer, Jürgen Krieger

**Affiliations:** 1 Department of Animal Physiology, Institute of Biology/Zoology, Martin Luther University Halle-Wittenberg, 06120 Halle (Saale), Germany; doreen.sander@zoologie.uni-halle.de (D.S.); thomas.karcher@bmglabtech.com (T.K.); 2 BMG Labtech GmbH, 77799 Ortenberg, Germany; 3 Advanced Light and Electron Microscopy, Centre for Biological Threats and Special Pathogens 4 (ZBS 4), Robert Koch Institute, 13353 Berlin, Germany; lauem@rki.de; 4 Microscopy Unit, Biocenter, Martin Luther University Halle-Wittenberg, 06120 Halle (Saale), Germany; gerd.hause@biozentrum.uni-halle.de; 5 Institute of Physiology, University of Hohenheim, 70599 Stuttgart, Germany; h.breer@uni-hohenheim.de

**Keywords:** olfaction, locust, sensilla, olfactory sensory neuron, support cell, odorant detection, sensory neuron membrane protein

## Abstract

**Simple Summary:**

The desert locust, *Schistocerca gregaria,* can form gigantic swarms of millions of individuals that devastate the vegetation of invaded landscapes. Locust food search, reproduction, and aggregation behaviors are triggered and controlled by complex olfactory signals. Insects detect odorants through different types of olfactory sensilla on the antenna that house olfactory sensory neurons and associated support cells, both of which express the proteins required for olfactory signaling. Among these proteins, two members of the CD36 lipid transporter/receptor family, named sensory neuron membrane proteins 1 and 2 (SNMP1 and SNMP2), are indicated to be of vital importance. Towards a better understanding of the role of the two SNMPs in the olfactory system of *S. gregaria*, we have analysed their antennal topography and subcellular localization using specific antibodies. The results indicate sensilla type- and cell type-specific distribution patterns of the SNMP proteins. SNMP1 was located in the receptive dendrites of subpopulations of olfactory sensory neurons as well as in the microvilli of associated support cells, suggesting a dual function of this protein, both in olfactory signal detection and in sensillum lymph maintenance, respectively. In contrast, SNMP2 was found solely in support cells and their microvilli membranes, suggesting a role limited to sensillum lymph recovery processes.

**Abstract:**

Insect olfactory sensilla house olfactory sensory neurons (OSNs) and supports cells (SCs). The olfactory sensory processes require, besides the odorant receptors (ORs), insect-specific members of the CD36 family, named sensory neuron membrane proteins (SNMPs). While SNMP1 is considered to act as a coreceptor in the OR-mediated detection of pheromones, SNMP2 was found to be expressed in SCs; however, its function is unknown. For the desert locust, *Schistocerca gregaria,* we previously visualized mRNA for SNMP1 in OSNs and SNMP2 mRNA in cells associated with OSN clusters. Towards an understanding of their functional implication, it is imperative to explore the cellular and the subcellular localization the SNMP proteins. Therefore, we have generated polyclonal antibodies against SNMP1 and SNMP2 and used fluorescence immunohistochemistry (FIHC) to visualize the SNMP proteins. We found SNMP1 in the somata and respective dendrites of all OSNs in trichoid sensilla and in subsets of OSNs in basiconic sensilla. Notably, SNMP1 was also detected in SCs of these sensilla types. In contrast, SNMP2 protein was only visualized in SCs of basiconic and coeloconic sensilla, but not of trichoid sensilla. Exploring the subcellular localization by electron microscopy using anti-SNMP1-ab and anti-SNMP2-ab revealed an immunogold labelling of SC microvilli bordering the sensillum lymph. Together our findings suggest a dual role of SNMP1 in the antenna of *S. gregaria*, in some OSN subpopulations in odor detection as well as in functions of some SCs, whereas the role of SNMP2 is limited to the functions of support cells.

## 1. Introduction

The desert locust, *Schistocerca gregaria*, is dreaded for its ability to form huge swarms of many millions of individuals that have devastating impacts on the vegetation and crops of invaded landscapes. In locusts, odorants originating from food sources and oviposition sites or released as pheromones from conspecifics are important olfactory cues that trigger various behaviors crucial for survival and reproduction [1,2,3,4] Odorant detection in locusts is accomplished by olfactory sensory neurons (OSNs) located in thousands of olfactory units, called sensilla, found mainly on the antenna [5,6] and in low numbers on mouthparts, i.e., the labial and maxillary palps [7,8]. On the antenna, locusts comprise three morphologically different sensilla with basiconic sensilla housing up to 50 OSNs, trichoid sensilla having 3 OSNs, and coeloconic sensilla bearing 4 OSNs. In each case, the OSNs are associated with several glia-like support cells [6]. In insects, both OSNs and support cells (SCs) express the proteins acting in the primary processes of odorant detection. While OSNs comprise olfactory receptors in their dendritic membrane that belong to the families of insect odorant receptors (ORs) and ionotropic receptors (IRs) [9,10,11,12], SCs express odorant binding proteins (OBPs) supposed to mediate the transfer of odorants through the sensillum lymph towards olfactory receptors [13,14,15]. In addition, so-called sensory neuron membrane proteins (SNMPs) are of critical importance in insect olfactory signaling [16,17,18].

SNMPs form an insect-specific lineage within the CD36 family of lipid/lipoprotein receptors and transporters [16,19]. CD36 proteins are characterized by two transmembrane domains and a large extracellular domain that is critical for ligand interaction [20,21]. Mammalian CD36 proteins, as well as non-SNMP family members in insects, have vital functions in the transport and reception of lipophilic compounds, lipoprotein scavenging, innate immune signalling, and cell adhesion [19,22,23,24]. Worth mentioning, in mice, a role of CD36 was reported in oleic acid detection by OSNs [25,26] as well as in sensing fatty acids by taste cells [27].

Insect SNMPs form a diverse gene family with variable numbers of SNMPs across species and orders. For example, only two SNMPs are found in *Drosophila melanogaster* [19] and *Schistocerca gregaria* [28], whereas 16 SNMPs were identified in the dung beetle *Onthophagus taurus* [29]. Based on phylogenetic relationships, insect SNMPs have been classified into four main groups (SNMP1—4) [29,30], with members of the SNMP1 and SNMP2 groups present in each species analyzed to date [31]. Consequently, studies on the expression and function of SNMPs in the olfactory system have concentrated on these two subtypes. The SNMP1 type has been discovered as protein expressed in the dendrites of pheromone-sensitive OSNs of the moth *Antheraea polyphemus* [32] and found be essential for a sensitive detection of lipophilic pheromones in *Drosophila melanogaster* [17,18] and heliothine moth species [33,34]. Additionally, a requirement for rapid activation and deactivation of pheromone-induced activity of OSNs has been reported [35]. Most recent data indicate binding of ligands to the large, tunnel-like ectodomain of SNMP1 [21] and colocalization in the dendritic membrane with the OR/odorant receptor–coreceptor (Orco) complex [30,36,37], supporting SNMP1 as further coreceptor possibly involved in the transfer of odorant molecules from OBPs to their cognate OR.

SNMP2s have been classified as a second SNMP type in moths, locusts, and other insect species reviewed in [31] and shares about 25–30% amino acid sequence identity to SNMP1. However, despite its name, sensory neuron membrane protein 2, SNMP2, exhibits a broad expression in support cells of olfactory sensilla [38,39], which are thought to control the composition of the sensillum lymph [40,41]. Given its expression in support cells and apparent evolutionary relationship to CD36 family proteins, SNMP2 has been suggested to function in clearing the sensillum lymph from lipophilic odorants or their degradation products [39,42]. However, so far only two immunohistochemical studies in the moths *Heliothis virescens* [39] and *Agrotis ipsilon* [38] provide a hint for localization of the protein in the microvillar protrusions of support cells bordering the sensillum lymph. 

In *Schistocerca gregaria*, the expression of SNMP1 and SNMP2 in the antenna has only been studied on the mRNA level. Using fluorescence in situ hybridization (FISH), SNMP1 transcripts were found in a subpopulation of the antennal OSNs, whereas SNMP2 expression was detected in cells surrounding OSN clusters, likely the support cells [28]. In addition, FISH experiments revealed co-expression of SNMP1 and certain members of the desert locust OR family in a subpopulation of OSNs located in basiconic sensilla and trichoid sensilla, but not in coeloconic sensilla [43]. Notably, SNMP1 was found co-expressed with 33 ORs of 83 ORs tested, suggesting that SNMP1 might function not only in the sensitive detection of pheromones but also of other important olfactory cues [44,45]. Together, the FISH experiments revealed a first picture of the expression of SNMP1 and SNMP2 on the mRNA level. However, with regard to the functions of SNMPs in the olfactory system of locusts, it is of critical importance to elucidate the cellular and subcellular localization of the SNMP proteins. Therefore, we set out to analyze the topography of SNMP subtypes in the antennae of *S. gregaria* on the protein level. Towards this goal, we have generated specific antibodies against the extracellular domains of the two proteins and used them in comprehensive fluorescent immunohistochemical approaches to explore the locust antenna. Furthermore, immunogold labelling experiments with transmission electron microscopy were performed to determine the subcellular localization of SNMP1 and SNMP2 in cells of olfactory sensilla. 

## 2. Materials and Methods

### 2.1. Animal Rearing

Desert locusts, *Schistocerca gregaria*, and migratory locusts, *Locusta migratoria*, were reared under crowded (gregarious) conditions as described in Seidelmann et al. [46]. Briefly, 100 to 150 individuals were kept in metal cages (50 × 50 × 50 cm) with metal grids at the bottom and at two sides. The photoperiod was 12L: 12D. The temperature was 34 °C during the day and 27 °C at night. The insects were fed with fresh wheat seedlings and flaked oats.

### 2.2. Bacterial Expression of S. gregaria SNMP Ectodomains (SgreSNMPecto)

The large ectodomains of SgreSNMP1 and SgreSNMP2 were expressed as His-tagged proteins using the pASK-IBA37plus expression system. The extracellular domains of the SNMPs were predicted with the THMMH 2.0 program [47] and amplified from plasmids containing the full-length coding regions using the following oligonucleotide primers: SgreSNMP1: 5′-AAG GAA TTC AAG CTC ATC TCC AGC CAG ATA-3′ and 5′-AAG CCC AAG CTT TTA GCC CTG CAT CCG GAA-3′; SgreSNMP2: 5′-AAG GAA TTC TTC CCC GCC ATT CTC ACC-3′ and 5′-AAG AAG CTT TTA CAT CGA CGC CCG CGC-3′. The PCR products were first cloned into the pGEM-T Easy Plasmid (Promega, Madison, WI, USA), excised from the vector by digestion with EcoRI-HF and HindIII (New England Biolabs, Ipswich, MA, USA) and subsequently ligated into the corresponding sites of the pASK-IBA37plus expression vector (IBA Lifesciences GmbH, Göttingen, Germany). The resulting SNMP1ecto/pASK-IBA37plus and SNMP2ecto/pASK-IBA37plus expression vectors were transformed into the *E. coli* strain MG1655. For SNMPecto expression, single colonies of the respective bacteria were inoculated in 100 mL LB medium with 100 µg/mL ampicillin. Bacteria were grown at 200 rpm and 37 °C until an OD_600_ between 0.6–0.7 was obtained. Then, expression was induced by adding 200 µg/L anhydrotetracycline. After incubation for 3 h, the bacteria were harvested by centrifugation at 5000 rpm for 15 min at 4 °C and resuspended in 6 mL of Tris-HCl buffer (50 mM Tris-HCl, 500 mM NaCl, 20 mM imidazole) supplemented with 1 mM phenylmethylsulfonyl fluoride (PMSF), 6 mM MgCl_2_, 4M urea, and 0.2 mg/mL lysozyme.

Successful bacterial overexpression of SNMP1ecto and SNMP2ecto proteins was verified by analyzing aliquots of induced and non-induced bacterial lysates by SDS-PAGE of on 12% gels and comparing protein patterns after Coomassie blue staining. Predicted sizes (including the His-tag and some vector-encoded amino acids) were 50.57 kDa for SNMP1ecto and 50.64 kDa for SNMP2ecto proteins and were evaluated by comparison to a protein molecular weight ladder (Thermo Scientific, Waltham, MA, USA).

### 2.3. Antibody Production

Proteins of induced bacteria overexpressing SNMP1ecto and SNMP2ecto proteins, respectively, were separated on parallel lanes by 12% SDS-PAGE and visualized by Coomassie blue staining. Intensive protein bands corresponding to the predicted size of the recombinant SgreSNMP1 and SgreSNMP2 proteins were excised out of the gels using a scalpel and collected to give an estimated amount of about 1.5 mg protein each. The proteins in gel fragments were used by a custom service (Davids Biotechnologie GmbH, Regensburg, Germany) to generate polyclonal antibodies against the ectodomains of SgreSNMP1 and SgreSNMP2 in rabbits using standard procedures and immunization for 63 days. The resulting antibodies, named anti-SNMP1-ab and anti-SNMP2-ab in the following, were purified using an antigen-specific affinity matrix.

### 2.4. Western Blot Analysis

For SDS-PAGE and Western blot analysis, 5 µg of total protein from bacterial lysates of induced and non-induced bacteria were separated in 12% gels. Two gels were prepared in parallel and either stained with Coomassie blue, or electroblotted with a semi-dry apparatus onto a PVDF membrane soaked in transfer buffer (25 mM Tris, 192 mM glycine, 20% methanol) at 200 mA for 1 h. The blotted membranes were then incubated for 1 h in TBST (100 mM Tris, 150 mM NaCl, pH 7.5 supplemented with 0.05% Tween 20) with 7% milk powder, followed by treatment with the primary antibodies, either anti-SNMP1-ab (1:7000) or anti-SNMP2-ab (1:7000), diluted in TBST with 3.5% milk powder overnight at 4 °C. Subsequently, the membranes were washed 3 times for 10 min with TBST, then incubated with goat-anti-rabbit alkaline phosphatase (ref. number 31346, Thermo Scientific) diluted 1:10,000 in TBST with 3.5% milk powder for 1h. After washing 3 times for 10 min each with TBST and 2 times for 10 min in substrate buffer (100 mM Tris-HCl, 100 mM NaCl, 5 mM MgCl_2_, pH 9.5), immune reactivity was detected by incubation in 0.0225% NBT (nitro blue tetrazolium) and 0.0175% BCIP (5-brom-4-chlor-3-indolyl phosphate) in substrate buffer.

### 2.5. Fluorescent Immunohistochemistry (FIHC)

Fluorescence immunohistochemistry (FIHC) was performed as previously described [39,48] with few modifications. Adult male and female *S. gregaria* and *L. migratoria* were removed from the stock cultures and cold anesthetized on ice. The antennae were carefully dissected and immediately embedded into Tissue-Tek O.C.T. freezing medium (Sakura Finetek, Alphen aan den Rijn, The Netherlands) at −20 °C. Cryosections (12 µM) of the samples were prepared with a Cryostar NX50 cryostat (Thermo Scientific) at −20 °C. Sections were thaw-mounted onto SuperFrost Plus slides (Thermo Scientific) and kept cold at −20 °C until encircling them using colourless ROTI^®^Liquid Barrier Marker (Carl Roth, Karlsruhe, Germany). Next, sections were fixed by incubation of the slides for 20 min at 4° C with 4% paraformaldehyde dissolved in phosphate buffered saline (PBS, 145 mM/L NaCl, 1.4 mM/L KH2PO4, 8 mM/L Na2HPO4, pH 7.4). Afterwards, the sections were rinsed at room temperature consecutively in PBS two times for 5 min, in PBS with 0.01% Tween20 for 5 min, in 50 mM NH_4_Cl in PBS for 5 min, and finally in PBS for 5 min. After incubating the samples in blocking solution (10% normal goat serum, 0.5% Triton-X100 in PBS) for 30 min at room temperature, the sections were incubated with the primary antibody diluted in blocking solution overnight at 4 °C in a humid box (anti-SNMP1-ab 1:500 on *S. gregaria* sections and 1:250 on *L. migratoria* sections; anti-SNMP2-ab 1:100). The slides were then washed three times for 5 min with PBS and subsequently treated with goat-anti-rabbit AF488-conjugated secondary antibodies (1:1000) (Jackson ImmunoResearch, Ely, Great Britain), goat-anti-HRP Cy3 (1:400) (Jackson ImmunoResearch), and DAPI (1:500, Thermo Fisher Scientific) diluted in PBS, for 1 h at room temperature in a humid box. Finally, the slides were washed two times for 5 min in PBS, once for 5 min in H_2_O, and then mounted in Mowiol solution.

### 2.6. Combined FIHC and Fluorescent In Situ Hybridization (FISH) 

Orco and SNMP1 antisense riboprobes were generated as described previously [8]. Briefly, specific primers were used to amplify coding sequences of SgreOrco (5′-CACTGGATGCTCGAGTACAGCGGCG and 5′-CGAGCTCTCTTCAATGAGCCTGTTG). The resulting products were cloned into pGEM-T Easy Plasmids (Invitrogen), which were subsequently used to generate digoxigenin-labelled Orco- and SNMP1-specific antisense RNAs using the T7/Sp6 RNA transcription system (Roche Diagnostics, Mannheim, Germany) as recommended by the manufacturer.

For combined FIHC/FISH experiments, antennae from *S. gregaria* were prepared, embedded, and sectioned into 12 µM slices as described above. After sectioning, the samples were treated for 20 min at 4 °C with 4% paraformaldehyde dissolved in PBS, washed in PBS for 5 min, and then incubated in 0.2 M HCl for 10 min. After washing the samples two times for 2 min in PBS, the slides were transferred into pre-hybridization solution (5× SSC (0.75 M NaCl, 0.075 M sodium citrate, pH 7.0) and 50% formamide) for 10 min. Next, each slide was covered with 130 µL hybridization solution (50% formamide, 25% H_2_O, 25% Microarray Hybridization Solution Version 2.0 (GE Healthcare, Freiburg, Germany)) containing either the ORCO or the SNMP1 antisense riboprobe. After placing a coverslip on top, slides were incubated in a humid box (50% formamide) at 60 °C overnight. The slides were then washed two times for 30 min each in 0.1× SSC at 60 °C. The sections were then washed with Tris-buffered saline (TBS; 100 mM Tris, 150 mM NaCl, pH 7.5) for 5 min at room temperature, which was followed by a blocking step in 1% blocking reagent (Roche Diagnostics, Mannheim, Germany) in TBS supplemented with 0.3% Triton X-100 for 30 min at RT. Afterwards, 130 µL of a mixture of anti-digoxigenin alkaline phosphatase-conjugated antibody (Roche Diagnostics) (1:500) and anti-SgreSNMP1-ab (1:200) diluted in 1% blocking reagent in TBS, 0.3% Triton X-100, were added on each slide. A coverslip was placed on top, and the slides were incubated at 4 °C overnight. The sections were then washed three times for 5 min in TBS supplemented with 0.05% Tween20 (TBST) and were transferred into 150 mM Tris-HCl solution (pH 8.3) for 5 min. For visualization of the digoxigenin-labelled probes, the Vector red alkaline phosphatase substrate kit (Vector Laboratories, Burlingame, CA, USA) was used according to the manufacturer. Briefly, 50 µL of each Vector red reagent (1,2, and 3) were diluted in 5 mL of 150 mM Tris-HCl (pH 8.3) to create the substrate solution that was applied to each section for 1 h at room temperature. The sections were then washed three times for 5 min in TBST, followed by an incubation with goat-anti-rabbitAF488 1:1000 and DAPI (1:500) diluted in TBST for 1h at room temperature. The sections were then washed two times for 5 min in H_2_O, then mounted in Mowiol. In combined FIHC/FISH experiments where FIHC was used to visualize neurons, the goat-anti-HRP Alex Fluor 647-conjugated antibody (Jackson ImmunoResearch) (1:200) was used in place of anti-SNMP1-ab and goat-anti-rabbitAF488.

### 2.7. Analysis of Antennal Sections by Confocal Microscopy

Sections from FIHC and FISH experiments were analysed on confocal laser scanning microscopes (LSM 880 and LSM780, Carl Zeiss Microscopy, Jena, Germany). Confocal image stacks of the fluorescence and transmitted-light channels were taken and used to generate either pictures representing single optical planes or projections of optical planes applying the ZEN software (Carl Zeiss Microscopy, Jena, Germany). Pictures were not altered except for adjusting the brightness or contrast for a uniform tone within a single figure.

### 2.8. Sample Preparation for Electron Microscopy and Immunogold Labelling

The antenna of *S. gregaria* were removed from the head, dissected into 1–2 mm long segments and transferred into the indentations of an aluminum specimen carrier (3 mm wide and 100 µm deep), which was filled with 1-hexadecene. Subsequently, the specimen carrier was covered by another aluminum specimen carrier (3 mm wide, flat) and high-pressure frozen with HPF01 compact (Engineering Office M. Wohlwend, Sennwald, Switzerland). Samples were freeze-substituted in acetone containing 0.5% glutaraldehyde, 5% distilled water for 24 h at −86 °C in an automatic freeze substitution system (AFS, Leica Microsystems, Wetzlar Germany). After increasing the temperature to −70 °C (1 °C/h), samples were incubated for 12 h at −70 °C, and then the temperature was raised to 0 °C (5 °C/h). After further 4 h incubation at 0 °C, the samples were briefly washed with acetone at room temperature, followed by washing twice for 5 min each with ethanol at room temperature. Subsequently, the antennal fragments were infiltrated with hard-grade LR White (Science Services, Germany) mixed 1:1 with ethanol for 30 min, followed by an ascending concentration of 2:1 (LR White:ethanol) for 30 min at room temperature and overnight incubation in pure LR White at 5–8 °C. After the samples were incubated in fresh LR White for additional 5 h at room temperature, the antenna were embedded in pure LR White and polymerized at 60 °C. Ultrathin sections (70 nm) were generated using an ultramicrotome (Ultracut S, Leica Microsystems) and collected on formvar coated nickel grids subsequently treated with blocking solution (1% acetylated BSA in PBS) for 30 min at room temperature then incubated with the primary antibodies (anti-SNMP1-ab 1:100, anti-SNMP2-ab 1:100) diluted in blocking solution over night at 4 °C. Afterwards, the samples were than rinsed 4 times for 5 min with blocking solution at room temperature, followed by treatment of the secondary anti-rabbit-antibody conjugated with 10 nm gold granules (G7402, Sigma-Aldrich, St. Louis, MO, USA) diluted 1:100 in blocking solution for 90 min at room temperature. After washing the samples four times for 5 min with distilled water, the grids were left to air dry and were ready for assessment with the transmission electron microscope (EM900, Carl Zeiss Microscopy) operating at 80 kV. The images were recorded using a Variospeed SSCCD camera SM-1k-120 (TRS, Moorenweis, Germany).

## 3. Results

### 3.1. Bacterial Expression of SNMP Ectodomains and Generation of Anti-SNMP Antibodies

**Figure 1:**
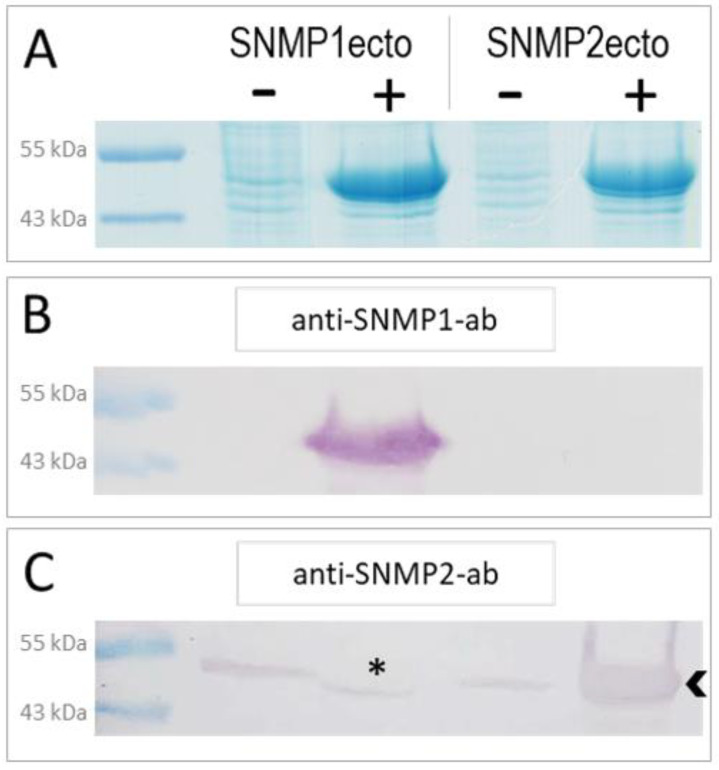
Heterologous expression of *S. gregaria* SNMP1 and SNMP2 ectodomains. (**A**) SDS-PAGE analysis of lysates from non-induced (−) and induced (+) SNMP1ecto and SNMP2ecto bacteria. In the induced samples, strong bands at the molecular weight predicted for the recombinant SNMP1ecto and SNMP2ecto proteins are visible at about 51 kDa indicating large amounts of expressed SNMP protein. (**B**) Western blot analysis of bacterial lysates as shown in (**A**) using the anti-SNMP1-ab showing intense labelling of a band only in induced SNMP1ecto bacteria. (**C**) Western blot analysis with the anti-SNMP2-ab showing labelling of a strong band in the induced SNMP2ecto bacteria (arrowhead). Weak labelling of bands in the other lanes indicate some cross reactivity of the antibody to endogenous bacterial proteins at about the molecular weight of SNMP2ecto, which results from using proteins from excised gel slices of induced bacteria for antibody generation. The asterisk denotes a possible displacement of a labelled band in the induced SNMP1ecto sample caused by the high amount of overexpressed protein.

In order to generate specific antibodies against *S. gregaria* SNMP1 and SNMP2, we have expressed the ectodomains of the respective proteins (Figure S1) in *E. coli* bacteria using an inducible expression system. The successful overexpression of the SNMP1ecto and SNMP2ecto proteins in induced bacteria was documented by SDS-polyacrylamide gel electrophoresis (PAGE) and Coomassie blue staining of total bacterial proteins from comparing induced (+) and not induced (−) samples (Figure 1A). Intense protein bands at the predicted molecular weight of the ectodomains of about 51 kDa were found only in the induced samples (Figure 1A). In a next step, the respective intense protein bands were excised from SDS-polyacrylamide gels and used to produce polyclonal antibodies against the SNMP1ecto and SNMP2ecto proteins in rabbits.

To assess the efficiency and specificity of the two newly created antibodies for SNMP1 and SNMP2, respectively, Western blot experiments with induced and uninduced samples from SNMP1ecto and SNMP2ecto bacteria were performed. The ectodomains of SNMP1 and SNMP2 share a sequence identity of approximately 34% (Figure S1) that is assumed to exclude a cross reaction of the corresponding antibodies. In accordance with this notion, anti-SNMP1-ab only detected an intense band of the correct size in the induced SNMP1ecto sample (Figure 1B), demonstrating the efficiency and specificity of the antibody for SNMP1. Similarly, anti-SNMP2-ab visualized a very strong band in the induced SNMP2ecto sample (Figure 1C). Faint bands were also visualized in the uninduced bacteria samples and the induced bacteria sample from SNMP1ecto. This is likely due to the detection of low amounts of native bacterial proteins that may have been present in the excised gel fragments used to generate the anti-SNMP2-ab. We also noticed that in the induced SNMP1ecto sample, the thin band runs slightly lower than in the non-induced samples, possibly due to displacement of reactive bacterial proteins by the huge amount of SNMP1ecto proteins, which most importantly are not detected by anti-SNMP2-ab (asterisk, Figure 1C). Since the presence of reactive bacterial proteins is very unlikely in antennal preparations, the observed cross reaction can be assumed to not disturb experiments with antenna. Together, the results indicate successful generation of specific antibodies against the ectodomains of *S. gregaria* SNMP1 and SNMP2, respectively, that were subsequently used for fluorescence immunohistochemical (FIHC) analysis.

### 3.2. Immunolocalization of SNMP1 Expression in the Antenna of S. gregaria

**Figure 2:**
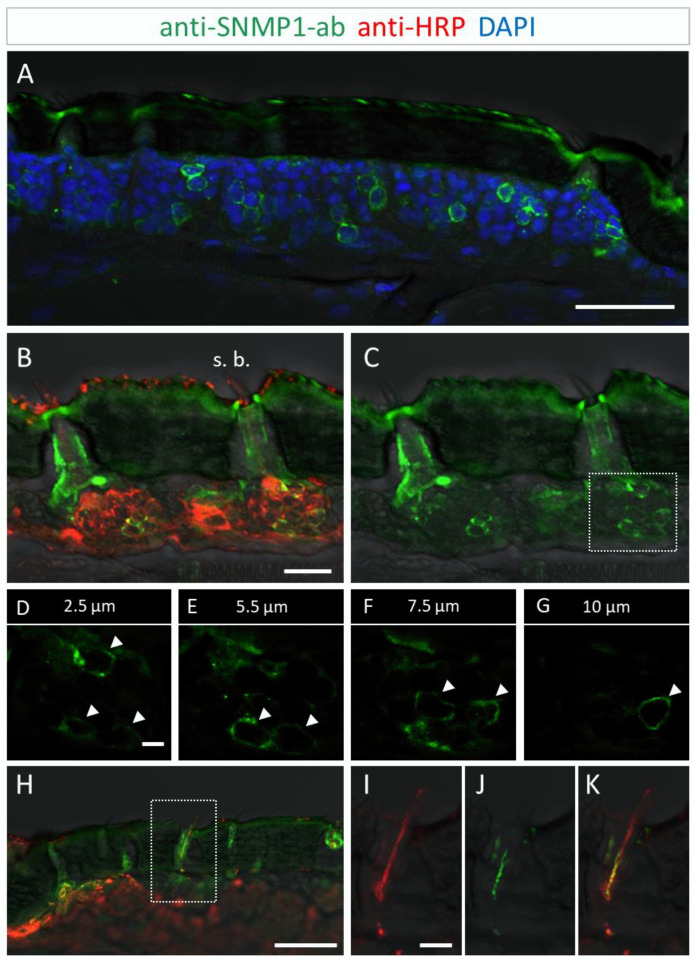
SNMP1 expression in a subset of OSNs in the antenna of *S. gregaria*. SNMP1-positive cells were visualized by FIHC in longitudinal sections using anti-SNMP1-ab (green). Neurons were identified by an anti-HRP-antibody (red) and nuclei were stained with DAPI (blue). (**A**) Distribution of multiple SNMP1 cells in an antennal segment. (**B**,**C**) Expression of SNMP1 (green channel) in a fraction of the OSNs (red channel) of basiconic sensilla (s. b.). (**D**–**G**) Higher magnifications of the OSN cluster boxed in (**C**) shown at different optical planes of a confocal z-stack; arrows denote SNMP1-positive neurons. (**H**–**K**) Localization of SNMP1 in OSN dendrites of a basiconic sensillum. (**I**–**K**) Higher magnifications of the area boxed in (**H**) showing the red (**I**, OSN) and green (**J**, SNMP1) channels separately or overlaid (**K**). The transmitted light channel was overlaid with fluorescent channels in (**A**–**C**,**H**–**K**). Scale bars: (**A**,**H**) = 50 μm; (**B**) = 20 μm; (**D**) and (**I**) = 5 µm.

To investigate the identity and distribution of the SNMP-expressing cells along the antenna of *S. gregaria,* we first conducted immunohistochemical approaches on tissue sections through the antenna with the newly generated anti-SNMP1-antibody. On longitudinal sections, anti-SNMP1-positive cells were found quite abundant within antennal segments and distributed in the tissue directly underneath the cuticle (Figure 2A).

Utilizing the anti-SNMP1 antibody in combination with an anti-HRP antibody for labelling of all neurons, we set out to simultaneously visualize the SNMP1-expressing cells and OSNs projecting to olfactory sensilla types. Comprehensive examinations of the anti-SNMP1-ab and anti-HRP immune reactivity revealed SNMP1 expression in the somata of distinct OSNs underneath basiconic sensilla (Figure 2B,C). Closer inspection of OSN clusters innervating a basiconic sensillum showed that multiple OSNs are SNMP1-positive, encompassing a subpopulation of the entire cluster (Figure 2D–G). This SNMP1 protein expression pattern is in accordance with previous in situ hybridization results [28,43,45]. Moreover, the SNMP1 protein was also visible in the dendrites of OSNs protruding into the shaft of basiconic sensilla (Figure 2H–K). In order to show the association of SNMP1 protein with OR-expressing OSNs, we performed combined fluorescence in situ hybridization (FISH) and FIHC experiments. Using a riboprobe for the OR-coreceptor Orco and the anti-SNMP1 antibody, SNMP1 immune reactivity was located in a subset of Orco-positive cells of a basiconic sensillum (Figure S2), demonstrating the expression of SNMP1 in OR-expressing OSNs. 

**Figure 3:**
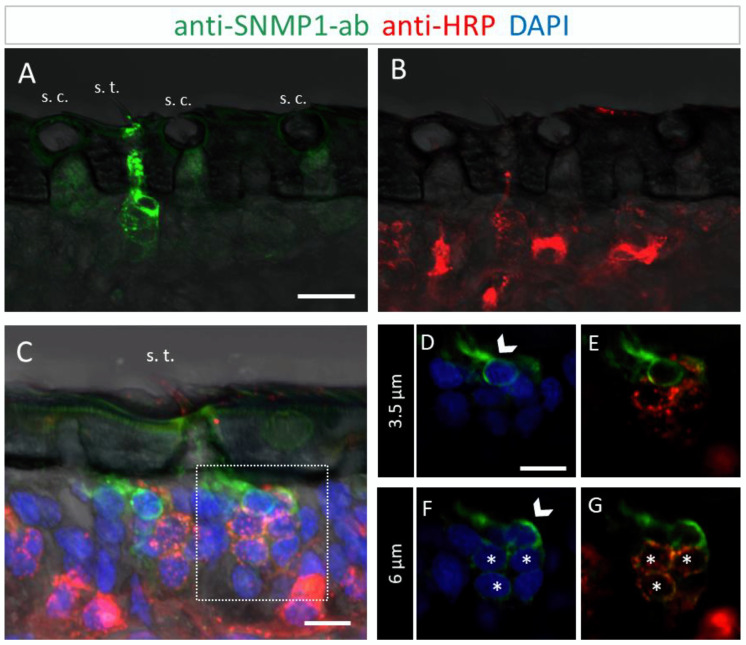
SNMP1 is localized in OSNs of trichoid sensilla (s. t.) but not of coeloconic sensilla (s. c.) in *S. gregaria*. In FIHC experiments with antennal sections, SNMP1-expressing cells (green) were visualized by anti-SNMP1-ab and neurons (red) by the anti-HRP-antibody. DAPI was used to stain nuclei (blue). (**A**,**B**) show anti-SNMP1-ab labelling of cells in trichoid, but not of coeloconic sensilla (**A**) and OSNs innervating the two sensilla types visualized in the red fluorescent channel (**B**). (**A**,**C**) trichoid sensillum from another treated section. (**D**–**G**) show a higher magnification of the area boxed in **C** showing the cell at the base in different optical planes and channels. (**D**,**E**) A non-neuronal, SNMP1-positive support cell is visible above an OSN cluster on a higher optical plane. (**F**,**G**) A second non-neuronal SNMP1-positive support cell as well as three OSNs are visualized in a deeper optical plane. Arrows indicate the SNMP1-positive support cells, asterisks the SNMP1-positive OSNs. The transmitted light channel was overlaid with the fluorescent channels in (**A**–**C**). Scale bars: (**A**) = 20 μm, (**C**,**D**) = 10 µm.

To further determine the topographic localization of SNMP1 protein, we next assessed the two other olfactory sensilla types on desert locust antenna. No anti-SNMP1-ab labelling of OSNs were found associated with coeloconic sensilla (Figure 3A,B). In contrast, intensive anti-SNMP1-ab immune reactivity was observed with cells underneath trichoid sensilla (Figure 3A). Closer inspection of the cells of trichoid sensilla indicated that unlike in the basiconic sensilla, in trichoid sensilla all innervating OSNs were positive for SNMP1 (Figure 3C,G, asterisks). However, the most intensive labelling was attributed to non-neuronal cells of trichoid sensilla that are closely associated with the OSNs and presumably represent support cells (Figure 3C–G). Analyzing different confocal planes of the tissue section exposed multiple support cells, which were positioned apical to a cluster of three OSNs that were anti-SNMP1-ab positive (Figure 3D,F, arrows). Analysis of another SNMP1-positive OSN cluster innervating a trichoid sensillum depicted up to three support cells that were labelled by anti-SNMP1-ab (Figure S3). In view of this finding, we reinspected basiconic sensilla. It was found that also here apical cells bordering OSN clusters, likely representing support cells of this sensillum type, were anti-SNMP1-ab-positive (Figure S4).

To further validate the observation that in *S. gregaria* SNMP1 is expressed in non-neuronal cells, we conducted experiments combining in situ hybridization and immunohistochemistry approaches using an antisense-SNMP1 riboprobe and the anti-HRP antibody to visualize neurons. As shown for a trichoid sensillum in Figure S5, we detected SNMP1 mRNA within the two depicted OSNs as well as in the adjacent non-neuronal support cells, corroborating the finding that the SNMP1 protein is present in both neuronal and non-neuronal cells in the antenna of *S. gregaria*.

To address the question of whether the expression of SNMP1 in OSNs and support cells is unique to the desert locust *S. gregaria* or also occurs in other locust species, FIHC experiments with anti-SNMP1-ab were performed on the section through the antenna of the migratory locust, *Locusta migratoria.* Since the SNMP1 ectodomains of the two locust species show about 90% sequence identity, cross-reaction of anti-SNMP1-ab with the SNMP1 of *Locusta migratoria* could be assumed. 

**Figure 4:**
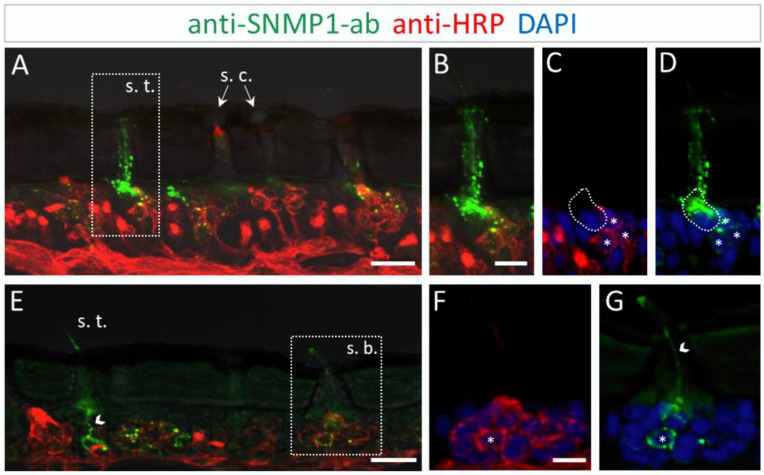
SNMP1 distribution in the antenna of *L. migratoria.* SNMP1-positive cells and neurons were visualized by FIHC on the longitudinal section using anti-SNMP1-ab (green) and anti-HRP-antibody (red), respectively. Nuclei were stained with DAPI (blue). (**A**) Shows that SNMP1 is localized in cells associated with the trichoid sensillum (s. t.). No labelling was found associated coeloconic sensilla (s. c.). (**B**–**D**) Displays the trichoid sensillum boxed in (**A**) in a higher magnification. (**B**) Green and red channels, (**C**) red and blue channels, and (**D**) green and blue channels. (**E**). Visualization of SNMP1-positive OSNs in trichoid and basiconic sensilla (s. b.). (**F**,**G**) Higher magnification of the basiconic sensillum boxed in (**E**) with (**F**) showing neuronal and (**G**) anti-SNMP1-ab labelling combined with nuclei staining. The asterisks denote SNMP1-positive neurons while the arrow heads indicate SNMP1-labelling of the dendrites. The encircled area indicates SNMP1 expression in a non-neuronal support cell. The transmitted light channel was overlaid with the fluorescent channels in (**A**,**B**,**E**). Scale bars: (**A**,**E**) = 20 μm; (**B**,**F**) = 10 μm.

The results of the FIHC experiments indicate that the localization of SNMP1 in *L. migratoria* antenna mirrored the antennal topography of SNMP1 in *S. gregaria*. Anti-SNMP1-ab labelling was observed in cells underneath trichoid sensilla (Figure 4A). When viewed in a higher magnification, it became apparent that not only are all three OSNs innervating the sensillum positive for SNMP1, but the most intensive labelling was detected in a neighboring support cell (Figure 4B–D).

Similar to *S. gregaria*, SNMP1 expression in *Locusta migratoria* was also found in distinct OSNs innervating basiconic sensilla (Figure 4E), whereas no signal was detected in coeloconic sensilla (Figure 4A,E). Like in *S. gregaria*, only a subset of OSNs in basiconic sensilla appeared to be SNMP1-positive. Furthermore, the anti-SNMP1-ab visualized dendrites protruding from SNMP1-positive OSN towards the sensillum shaft (Figure 4G). Taken together, these results indicate the distinctive expression pattern of SNMP1 in locust antennae, with SNMP1 found not only in OSNs but also in support cells.

### 3.3. Immunolocalization of SNMP2 Expression in the Antenna of S. gregaria

**Figure 5:**
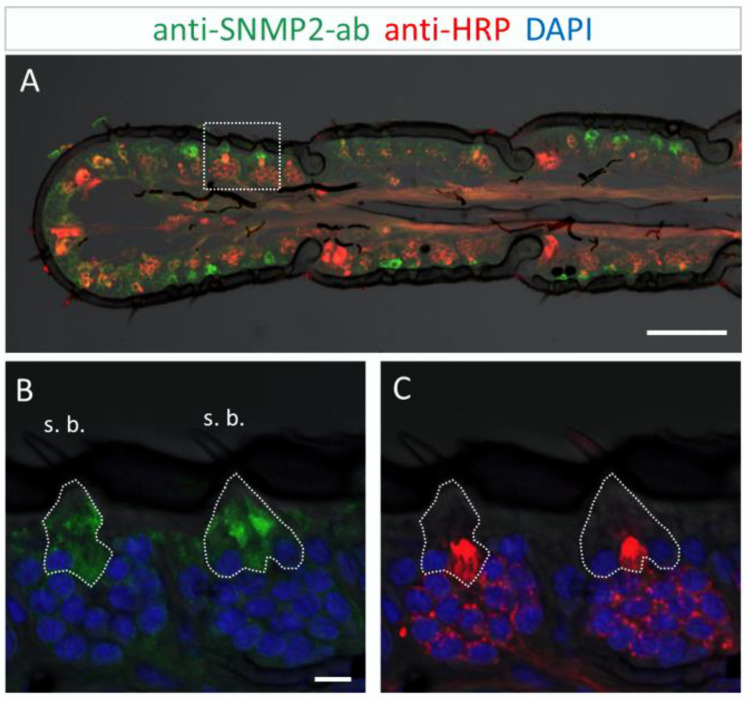
SNMP2 expression in cells of the *S. gregaria* antenna. SNMP2-positive cells were visualized by FIHC in the longitudinal section using anti-SNMP2-ab (green). Neurons were visualized by anti-HRP-antibody (red) and nuclei by staining with DAPI (blue). (**A**) Topography of SNMP2-expressing cells in multiple antennal segments. (**B**,**C**) Displays the area boxed in (**A**) in a higher magnification and different channel. (**B**) Shows the green and blue channels, demonstrating the expression of SNMP2 in support cells (encircled) underneath basiconic sensilla (s. b.). (**C**) Shows the red and blue channels, displaying the clusters of OSNs housed in the basiconic sensilla. Neuronal labelling within the encircled area corresponds to the dendrites of OSNs passing by support cells on the way to the hair shaft. In all images, the fluorescent channels were overlaid with the transmitted light channel. Scale bars: (**A**) = 100 µM; (**B**) = 10 µM.

In order to analyze the cellular expression pattern of the SNMP2 protein in *S. gregaria* antennae and to compare it to SNMP1, we next conducted FIHC experiments using anti-SNMP2-ab and anti-HRP antibodies. As exemplarily demonstrated on the longitudinal section shown in Figure 5A, large numbers of anti-SNMP2-ab-positive cells were visible across different antennal segments below the cuticle. In addition, the anti-HRP antibody visualized numerous clusters of neuronal cells (OSNs) that were not labeled by the anti-SNMP2 antibody.

**Figure 6:**
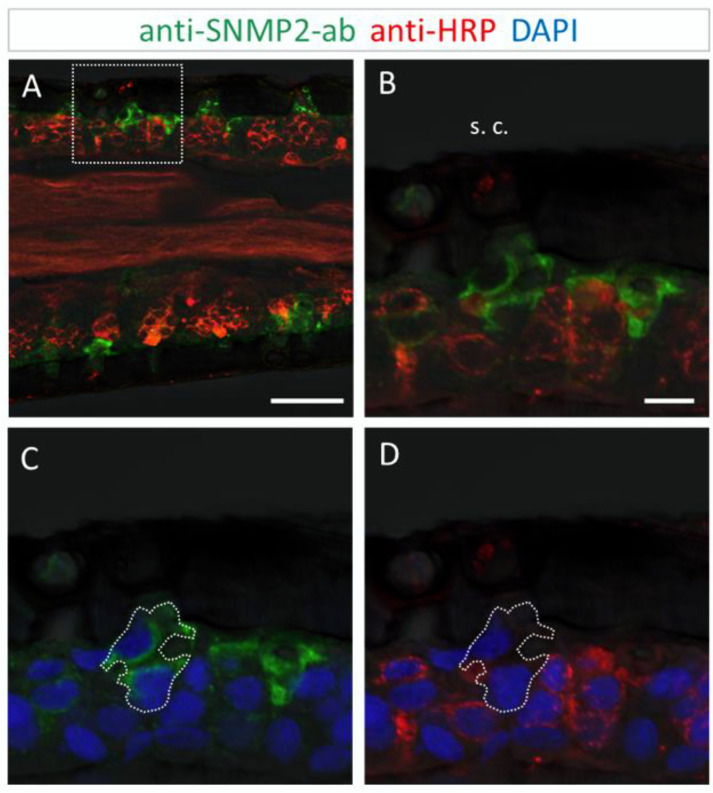
SNMP2 is expressed in non-neuronal cells of coeloconic sensilla in the antenna of *S. gregaria*. Longitudinal sections were assessed by FIHC using anti-SNMP2-ab to identify SNMP2-expressing cells (green) and anti-HRP to visualize OSNs (red); sections were stained with DAPI to visualize nuclei (blue). (**A**–**D**) Localization of SNMP2 in multiple non-neuronal cells. (**B**–**D**) Show the boxed area in (**A**) in a higher magnification. (**B**) Red and green fluorescent channels, (**C**) green and blue channels, and (**D**) red and blue channels. The encircled area denotes SNMP2-positive labelling of support cells under a coeloconic sensillum (s. c.). In all images the fluorescent channels were overlaid with the transmitted light channel. Scale bars: (**A**) = 50 µM; (**B**) = 10 µM.

This was more clearly seen upon closer inspection of basiconic sensilla at higher magnification (Figure 5B,C). While no anti-SNMP2-ab immunoreactivity was detected within any of the OSN somata, large cells situated beneath the sensillum shaft were found positive for SNMP2 (Figure 5B). These observations suggest that support cells are directly associated with the entire cluster of OSNs, seemingly enveloping the base of bundled dendrites projecting into the sensillum (Figure 5C). Attempts to assess the localization of SNMP2 protein in trichoid sensilla using the anti-SgreSNMP2 antibody showed no clear results. Occasionally, very weak labeling of non-neuronal cells hardly above background staining was observed. In contrast, intensive immunolabelling by the anti-SgreSNMP2-ab was obtained for cells directly beneath coeloconic sensilla (Figure 6). Anti-HPR counter staining for neurons confirmed that, similar to basiconic sensilla (Figure 5), the anti-SNMP2-ab-positive cells of coeloconic sensilla are non-neuronal cells (Figure 6B,C), most likely the support cells associated with the OSNs. Attempts to label SNMP2-expressing cells in the antenna of the closely related species *L. migratoria* with the anti-SNMP2-ab did not lead to conclusive results.

### 3.4. Subcellular Localization of SNMP1 and SNMP2 within Sensilla

In order to evaluate the subcellular localization of SNMPs, we analyzed their expression topography on ultrathin sections on the antenna of *S. gregaria* by transmission electron microscopy (TEM). In the immunogold labelling experiments, we obtained clear results for both SNMP types and the abundant basiconic sensilla, whereas our attempts to clarify their subcellular localization in trichoid and coeloconic sensilla were not successful and would require further investigations.

**Figure 7:**
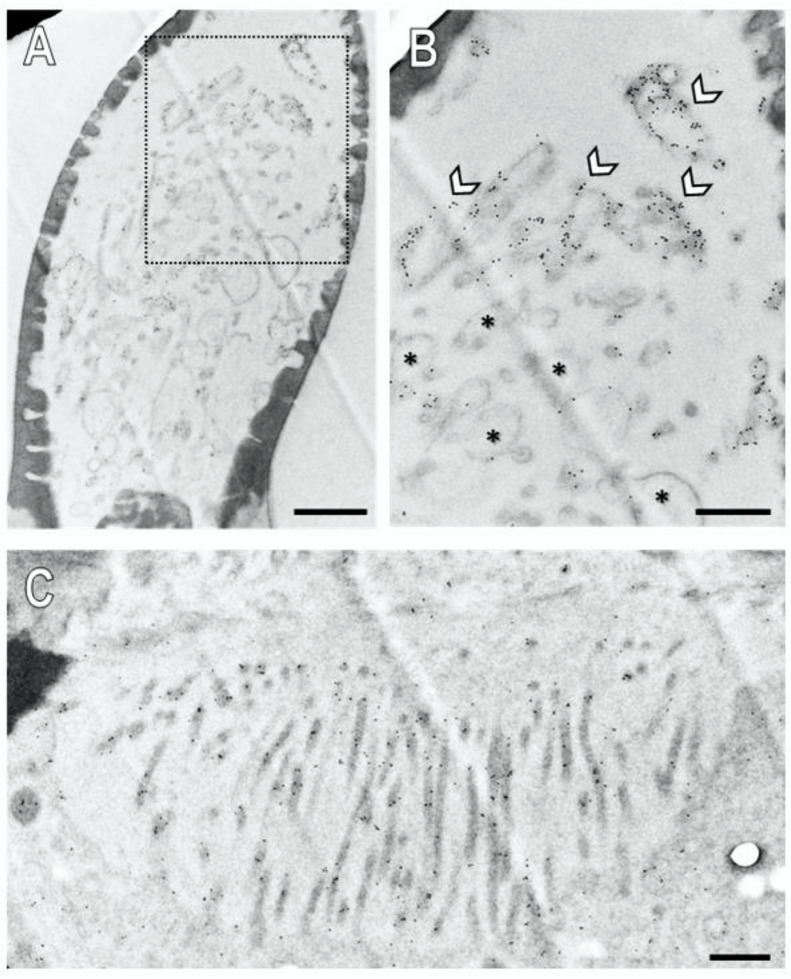
Subcellular localization of SNMP1 within a basiconic sensillum of the *S. gregaria.* Ultrathin sections were assessed by transmission electron microscopy after treatment with anti-SNMP1-ab and a secondary anti-rabbit antibody coupled with colloidal gold. (**A**) Shaft of a basiconic sensillum with multiple dendritic structures in the lumen. (**B**) Higher magnified image of the area boxed in (**A**). Intense immunogold labelling is observed associated with the membranes of a subset of dendritic structures (arrows). The asterisks denote dendritic structures with little to no immunogold labelling. (**C**) Microvilli structures of support cells at the base of the sensillum. Significant immunogold labelling was found associated with the microvilli including their membranes. Scale bars: (**A**) = 1 µm; (**B**,**C**) = 500 nm.

Assessment of a longitudinal section through a basiconic sensillum treated with the anti-SNMP1 antibody showed multiple dendritic structures in the sensillum shaft in accordance with multiple OSNs housed in this sensillum type that comprise branched dendrites (Figure 7A). At higher magnification, it became apparent that the membranes of some of the dendritic structures were intensively immunogold labelled (Figure 7B, arrows; Figure S7B), whereas others showed no or only little labelling (Figure 7B, asterisk). Corresponding control experiments omitting the primary SNMP antibodies showed no labelling demonstrating no unspecific binding of the secondary antibody to structures within a sensillum (Figure S6). Together, the results show that SNMP1 is located only in a subset of the dendritic structures within a basiconic sensillum. This finding is in line with the expression of SNMP1 in only a subset of the OSNs in basiconic sensilla (Figure 2).

Our FIHC results on the light microscope level have indicated expression of SNMP1 also by support cells of basiconic sensilla (Figure S4). In accordance with this finding, we analysed the base of the basiconic sensilla (Figure 7C and Figure S7C) and found anti-SNMP1 immunogold labelling associated with microvilli bordering the sensillum lymph. These membraneous microvilli structures form the apical region of the support cells that are situated directly beneath the sensillum lumen. These findings further substantiate the expression of SNMP1 in support cells and localize the protein in non-neuronal microvilli protruding towards the sensillum’s base. 

**Figure 8:**
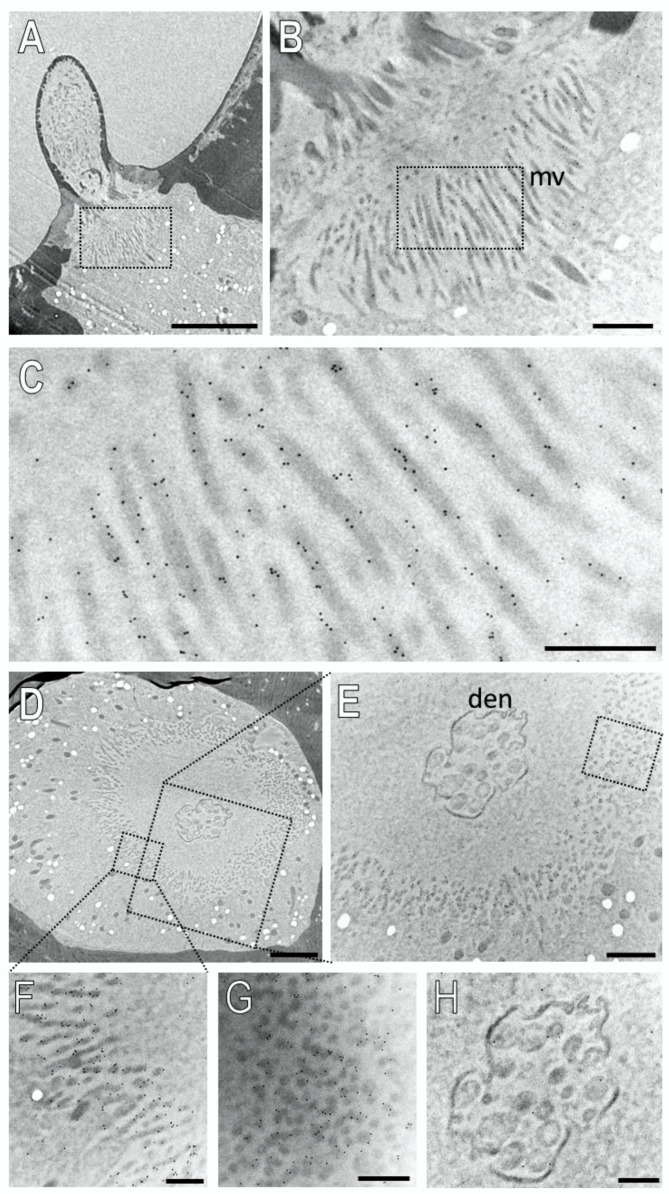
SNMP2 is localized in microvilli of support cells of basiconic sensilla. Ultrathin sections were treated with anti-SNMP2-ab in immunogold labelling experiments and assessed by transmission electron microscopy. (**A**) Longitudinal section through a basiconic sensillum. (**B**) Higher magnification of the region boxed in (**A**), showing microvilli structures (mv) at the base of the sensillum. (**C**) Higher magnified image of the region boxed in (**B**), displaying clear anti-SNMP2-ab immune reactivity associated with microvilli. (**D**) Cross section through the base of another basiconic sensillum. (**E**) Higher magnified image of the large box depicted in (**D**). A higher magnification of the smaller box in (**D**) is shown in (**F**), indicating strong immunogold labelling of microvilli structures. (**G**) is a higher magnification of the microvilli boxed in (**E**), depicting SNMP2-labelling associated with transversely cut microvilli. (**H**) is a higher magnification of the dendrites (den) shown in (**D**,**E**), indicating only low levels of background signal. Scale bars: (**A**) = 5 µm; (**B**,**E**) = 1 µm; (**D**) = 2 µm; (**C**,**F**–**H**) = 500 nm.

Next, we examined the subcellular localization of SNMP2 in basiconic sensilla. Figure 8 shows the result of an ultrathin section consecutive to that shown in Figure 7. Intensive anti-SNMP2-ab immunogold labelling was found associated with microvilli structures originating from support cells at the base of the sensillum shaft (Figure 8A–C); no labelling of dendritic structures within the sensillum lumen was observed. To assess the anti-SNMP2-ab labelling of the microvilli in relation to the sensillum’s lumen, a cross-section through the base of a basiconic sensillum innervated by dendrites of multiple OSNs was inspected in more detail (Figure 8D,E). Again, inspection of the dendritic bundle in the center of the sensillum at a higher magnification showed only single gold grains, which most probably represent background labelling (Figure 8H). In contrast, vast anti-SNMP2-ab immunogold labelling was found associated with microvilli structures of support cells that completely surround the internal sensillum lumen (Figure 8E). Analyzing the microvilli structures at a higher magnification demonstrates that the SNMP2 protein is localized at the direct interface between the support cell and the lymph space (Figure 8F,G). Taken together, these results clearly indicate that SNMP2 protein is solely present in non-neuronal support cells and localized in the microvilli that protrude directly into the sensillum lymph filling the sensillum shaft.

## 4. Discussion

In this study, we examined the topography and subcellular localization of SNMP1- and SNMP2-protein in the antennae of the desert locust *S. gregaria* using specific antibodies targeting the ectodomains of the respective proteins.

Performing FIHC-experiments using the anti-SNMP1 antibody resulted in visualization of SNMP1 protein in populations of Orco-positive OSNs including their dendrites, which project into trichoid and basiconic sensilla. These results confirm our previous findings visualizing cells with mRNA for SNMP1 by in situ hybridization experiments [28,45] but reveal the cellular and subcelluar localization of the SNMP1 protein. Immunolabeling for SNMP1 was not detected in OSNs of coeloconic sensilla, which in *S. gregaria* express IRs [49]. Similarly, a co-expression of SNMP1 with ORs but not with IRs in OSNs has been reported for *D. melanogaster* [17] and *Microplitis mediator* [50]. Together, these results further substantiate the notion that SNMP1 expression is confined to OR-expressing OSNs, which appears to be a conserved pattern across different species. 

Using anti-SNMP1-ab, we found all OSNs innervating a trichoid sensillum to be SNMP1-positive, while in basiconic sensilla only a subset of OSNs express SNMP1. The same result was obtained for the related species, *L. migratoria,* demonstrating a conserved distribution pattern of SNMP1-expressing OSNs across olfactory sensilla of locusts. Hence, the data indicate that basiconic and trichoid sensilla of locusts not only differ in their morphology, but also in the molecular equipment of their OSNs, suggesting that all OSNs of trichoid sensilla employ SNMP1 in odor detection. An expression of SNMP1 in all OSNs of trichoid sensilla has also been reported for *D. melanogaster* [17] and females of the moth *Heliothis virescens*. In contrast, in males of *H. virescens,* only one of the 2–3 OSNs housed in the trichoid sensilla were found SNMP1-positive [39,51], demonstrating that SNMP1 expression is not generally associated with the OSNs of trichoid sensilla.

Various studies on fly [17,18,35] and moth species [33,34] have shown that SNMP1-positive OSNs of trichoid sensilla are associated with the sensitive detection of pheromones, suggesting that OSNs in locust trichoid sensilla may also serve in pheromone detection. In accordance with this notion, previous single sensillum recordings from *S. gregaria* trichoid sensilla revealed OSNs that are tuned to a possible sex pheromone component [52]. However, although all OSNs in trichoid sensilla of *L. migratoria* express SNMP1, distinct OSNs of trichoid sensilla express the receptor type OR3 that is activated by various non-pheromonal odorants [53]. Hence, expression of SNMP1 in trichoid OSNs may not necessarily be linked to pheromone detection. 

In both assessed locust species, we found that only a subset of the antennal OSNs innervating basiconic sensilla were SNMP1-positive indicative for SNMP1-dependent and SNMP1-independent mechanisms of signal processing in basiconic OSNs. This notion is in accordance with our recent finding that from the 80 OR types expressed in basiconic OSNs of *S. gregaria,* only 30 OR types are co-expressed with SNMP1 [43,45]. 

Single sensillum recordings from locust basiconic sensilla, which house up to 50 OSNs, revealed sensilla responses to a variety of general odorants (plant volatiles), odorant mixtures representing adult and nymphal body odors, as well as to verified pheromones [2,52]. Due to the large number of neurons, an assignment of odor responses to a distinct OSN is unfortunately not possible, but the results indicate that the ensemble of OSNs in basiconic sensilla allows a contribution in the detection of specific pheromones as well as general odorants. Given the rather broad expression of SNMP1 in populations of basiconic OSNs expressing many different OR types, it is therefore conceivable that in locusts the neuronal SNMP1 is not an obligate co-receptor required for a sensitive response of OSNs to pheromones. Instead it may also act in a sensitive detection of behaviorally important non-pheromonal odorants originating from various biological sources, such as preferred food plants or oviposition sites [1,4] as indicated for *D. melanogaster* SNMP1 that was found essential for sensitive pheromone detection [17,18], but was also shown to be required for the proper response of OR83c-expressing OSNs to the plant-derived odorant farnesol [54].

Notably, SNMP1 was also present in non-neuronal SCs of trichoid and basiconic sensilla from *S. gregaria* as well as *L. migratoria.* Expression of SNMP1 in SCs was not noticed in previous in situ hybridization experiments possibly due to a missing neuronal counter stain [28]. In trichoid sensilla, we detected up to three SNMP1-positive SCs adjacent to three SNMP1-positive OSNs. This SNMP1 expression pattern resembles the situation in *D. melanogaster*, where the SNMP1-positive OSNs of trichoid sensilla are associated with several SNMP1-expressing SCs [17]. Together, the current data suggest that SNMP1 plays a distinct role in OSNs and SCs in the olfactory system of locusts and flies. In contrast, in moths, SNMP1-expression in trichoid sensilla was found to be restricted to OSNs, whereas SNMP2 was exclusively expressed in SCs [39,55,56], suggesting some functional specialization of moth SNMP1.

In comparison to SNMP1, the SNMP2 protein was solely detected in SCs. Strong labelling was obtained for SCs in basiconic and coeloconic sensilla, the two most abundant sensillum types on the antenna of *S. gregaria*. Despite considerable efforts, we could not visualize unambiguous SNMP2 immune reactivity in SCs of the trichoid sensilla. Whether this was due to very low levels of SNMP2 protein or due to the absence of the SNMP2 protein in trichoid SCs remains elusive. In this context, it is interesting to note that SNMP1 is clearly expressed in SCs of trichoid sensilla. It is therefore tempting to speculate that in SCs of trichoid sensilla, SNMP1 has replaced SNMP2 and may fulfill a function similar to SNMP2 in SCs of other sensilla types.

In accordance with the notion that the two SNMP types fulfil similar functions in SCs, the immunogold-labelling experiments demonstrate a similar subcellular localization for *S. gregaria* SNMP1 and SNMP2. Both proteins were located to the membranous microvilli of SCs directly bordering the lymph-filled sensillum lumen; these microvilli are considered as a site of high transmembrane transport and exchange activity [41]. Consistently, FIHC experiments have indicated that in the antenna of the moth *Heliothis virescens,* SNMP2 was located in the most apical region of the SCs [39], suggesting conserved roles of SNMPs in SC across insect orders and a function at the interface to the sensillum lymph. While the specific role of non-neuronal SNMPs awaits further investigation, based on the function of other CD36 family members it is conceivable that SNMP2 may operate as transporter involved in the maintenance of the sensillum lymph. Mammalian CD36 proteins and insect members of the CD36 family outside of olfaction, such as NinaD and Santa Maria are vital for the capture and uptake of lipophilic molecules including fatty acids into cells [57,58,59,60]. Accordingly, SNMPs in the microvilli membrane of SCs may play an integral role in capturing lipophilic molecules (odorant, pheromones, or their degradation products) emerging in the sensillum lymph during olfactory processes and the subsequent translocation of the “waste product”, either by themselves or in cooperation with other transport proteins, into the SC for further disposal.

In view of a putative function in the lymph clearance, it is interesting to note that SNMP1 is expressed in SCs associated with OSNs that express SNMP1. Thus, SNMP1 in SCs of trichoid and basiconic sensilla may be involved in the clearance of “waste products” resulting from odorants that are detected by OSNs, which use SNMP1 as a co-receptor. Correspondingly, SNMP2 may function in SCs of basiconic and coeloconic sensilla in order to eliminate “waste products” arising from SNMP1-independent odorant detection processes. 

In conclusion, we have demonstrated that SNMP1-protein is present in populations of OSNs and in non-neuronal supporting cells of olfactory sensilla on the locust antenna.

An assessment of the subcellular localization revealed SNMP1 protein was present in the dendritic membrane of OSNs as well as in the microvilli of the SCs. In contrast, SNMP2 protein was exclusively located in the microvilli of the SCs. Thus, in olfactory sensory neurons, SNMP1 may function as coreceptor involved in signal recognition, whereas in non-neuronal cells both SNMP subtypes may be involved in the maintenance of the sensillum lymph and, due to its homology to CD36 proteins might be involved in the uptake of lipophilic “waste products”.

## Data Availability

Data is contained within the article or supplementary material.

## Supplementary Materials

The following file contains the supplementary Figures S1–S7 and can be downloaded at: https://www.mdpi.com/article/10.3390/insects13070579/s1; Figure S1: Alignment of the amino acid sequences of *S. gregaria* SNMP 1 and SNMP 2.; Figure S2: SNMP 1 is expressed in a subset of Orco positive OSNs in basiconic sensilla.); Figure S3: FIHC experiment showing that SNMP 1 is expressed in OSNs and the associated support cells of a trichoid sensillum; Figure S4: SNMP 1 is expressed in distinct OSNs and support cells of a basiconic sensillum in *S. gregaria*; Figure S5: SNMP 1 is expressed in support cells and OSNs of a *S. gregaria* trichoid sensillum; Figure S6: Assessment of secondary antibody binding to ultrathin sections of the *S. gregaria* antenna; Figure S7: Localization of SNMP 1 within dendrites of OSNs an microvilli structures of support cells in a basiconic sensillum of *S. gregaria*.
