# Supplementary material for: The Sensilla-Specific Expression and Subcellular Localization of SNMP1 and SNMP2 Reveal Novel Insights into Their Roles in the Antenna of the Desert Locust Schistocerca gregaria
Source: Insects. 2022 Jun 25;13(7):579. doi: 10.3390/insects13070579 (PMC9317141; doi:10.3390/insects13070579)
Supplement: Supplementary file 1 [file insects-13-00579-s001.zip › insects-1776531-supplementary.pdf]

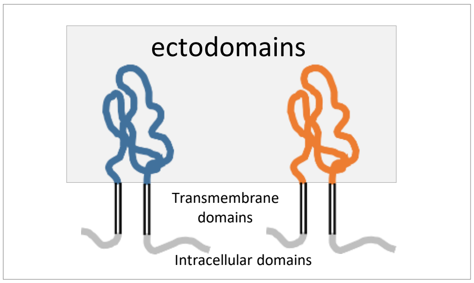


```
SgreSNMP1        1 -----------MQLPVGLAAGGGGVFFMAVVAGWYGMPKLISSQIASGLA     39
                              ..|..|||| |..:..:|:|..|...|.:::::|...:.
SgreSNMP2        1 MLSARVCGCGRRGLWWGLAA-GAALLAVALVLRWAAFPAILTAKIKQAVQ     49

SgreSNMP1       40 LKKGSDIRQMWSNFSDPIDFRVYVLNLTNPEAVHRGEKPIVQEIGPYFYE     89
                   |..||...:.:.....|:.::||:.|:|||:.|.:|.||::|::|||.||
SgreSNMP2       50 LHDGSPAMERFVQLPQPLLYKVYLFNVTNPDEVEQGAKPVLQQVGPYVYE     99

SgreSNMP1       90 EYKQKVKLRDHKEDDTVSYNNKITWLFNQGKSAPGLTGDELVTLPHPLLL    139
                   |:::::..: ....:.::.|..:.|:.|:..:| |||:.|:..|..:.::
SgreSNMP2      100 EWRRRRDV-TRMANGSLDYRLETTYHFSPERS-PGLSEDDEFTYLNVVMV    147

SgreSNMP1      140 GLLLTLERDKPGMLALVNKAIPPLFRKPESIFVTAPVRNFLFDGI-VINC    188
                   |:::.:..|...:|::|...:..|......:|..|..|..|:.|: .::|
SgreSNMP2      148 GIVVQVSEDYSSLLSMVEPVLSELVPGGAQLFQRASARQLLWSGVPTVDC    197

SgreSNMP1      189 TVTDFSAKALCTGLKKE---AKELKREGDNFFFSFFGHKNGTVDAGRLRV    235
                   .....:...|..|....   |...:.|...:.|||||.|||| .....||
SgreSNMP2      198 RGNLSAVATLACGALPSLLPATVQQTEPGVYVFSFFGFKNGT-SKQWWRV    246

SgreSNMP1      236 KRGIQNIDDLGRVVAFNGEPKMSAW--RGDPCNDLRGTDSTIFPPFRDPK    283
                   ..|:::::..||.|::::...::..|  ...|||::|||||||:||||..|.
SgreSNMP2      247 DSGVEDVRTLGSVISYDNSSRLKVWSPSNSPCNEIRGTDSTLFPPFITPN    296

SgreSNMP1      284 EPIVAFGPDLCLSLGANWERKAEYMGVPGNRYTAELPDMKGNPEHHCYCP    333
                   :.|..|..|:|.|:.|.:||:.:..||.|.|:.|....::.....:.|.||
SgreSNMP2      297 DTIYIFAHDICRSMHAEYEREQDVSGVHGLRFVASGSLLRRGGPNACTCP    346

SgreSNMP1      334 TEQTCLEKGTLDLSPCAGAPVIATLPHFYLASETYLQTVSGLQPTKENHE    383
                    :..||..|.:.:..|..||:..:.||||.||..|||...||.|.||.||
SgreSNMP2      347 -DGRCLATGAISVRECFRAPIAVSFPHFYQASPEYLQYAEGLSPNKELHE    395

SgreSNMP1      384 LFMVFESTTGSPMEARKRLQFNMFLHKINKIDLLANVPYALMPLIWVEEG    433
                   .|:|.|..||:|:...|||||||...:::::..|.||...|.||:|||||
SgreSNMP2      396 TFVVIEPETGTPLVGAKRLQFNMKAVRVSQVPALRNVSDGLFPLLWVEEG    445

SgreSNMP1      434 LALEEKYVSTLRMLFRMQGIMSGVKWTLMAVGMGMAGAGGYLHFKRRKEL    483
                   :.||||.:|.:|.|:..:..|.||.|.::|||:    |.......|..:.
SgreSNMP2      446 VELEEKQLSQVRALYVARASMGGVAWAVLAVGV----AALLFCAVRLAKA    491

SgreSNMP1      484 VVGPAEPKKVVAGHDTTGHPIRLESSHSRY--------------------    513
                   .|.......:....|.|..:.:.:..:.|
SgreSNMP2      492 RVAERNRSLSLEKGVTAGGKLSVPTLGAAYPESATKRPSPPAAAPAASTA    541

SgreSNMP1      514 ----------     513

SgreSNMP2      542 PAAPVDATHF     551
```

Figure S1. Alignment of the amino acid sequences of *S. gregaria* SNMP1 and SNMP2. Pairwise sequence alignment were conducted with EMBOSS Needle. Light grey underlined sequences represent intracellular regions of the proteins while black underlined areas denote predicted transmembrane domains. The region indicated by the grey background show the extracellular domains of the proteins used to generate antibodies. The ectodomains of SNMP1 (blue) and SNMP2 (orange) have a sequence identity of 33.7%.

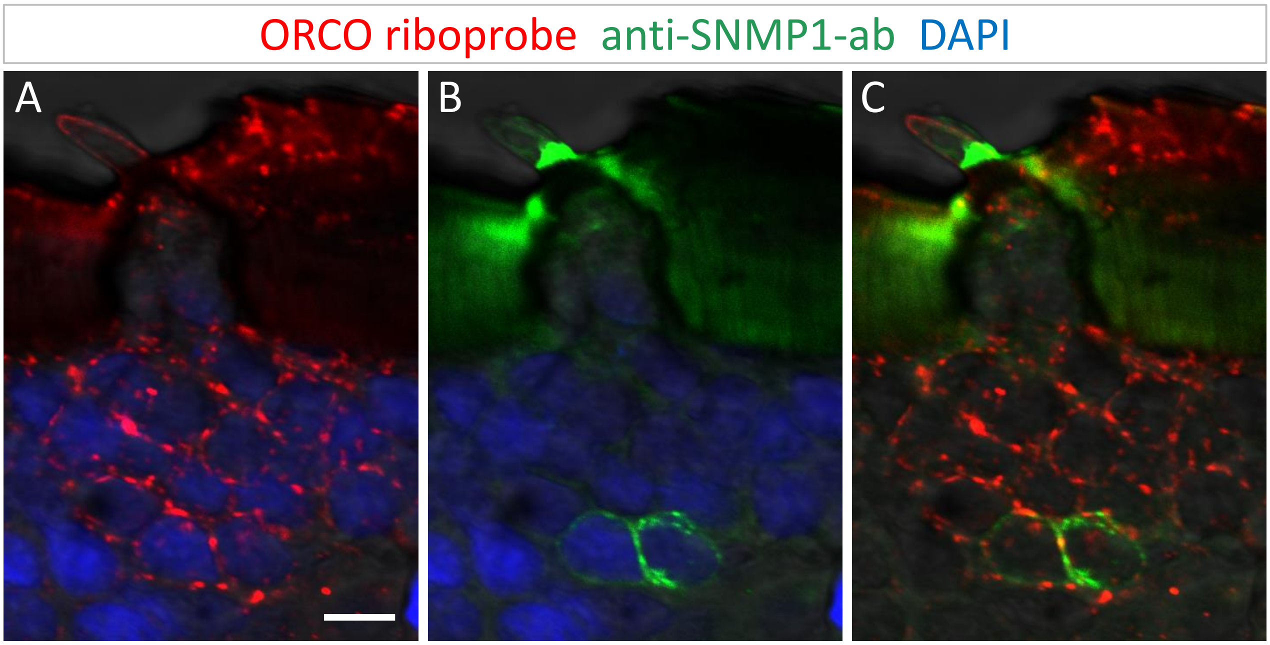

Figure S2. SNMP1 is expressed in a subset of Orco-positive OSNs in basiconic sensilla. In combined FISH/FIHC, Orco-positive cells were visualized using an antisense-Orco riboprobe (red), whereas SNMP1-positive cells were detected using anti-SNMP1-ab (green). Nuclei were stained with DAPI (blue). **A** red and blue channel. **B** green and blue channel. **C** merged depiction of the green and red channel. In all images, the fluorescent channels were overlaid with the transmitted light channel. Scale bar = 10 µm.

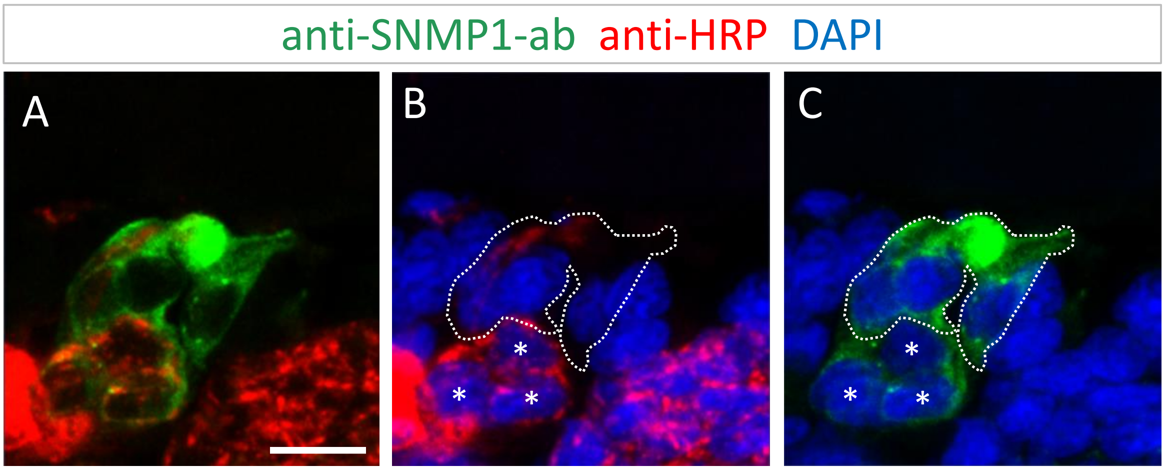

Figure S3. FIHC experiment showing that SNMP1 is expressed in OSNs and the associated support cells of a trichoid sensillum. The anti-SNMP1-ab labelling is shown in green. Neurons are labelled with anti-HRP and shown in red while the nuclei are stained with DAPI shown in blue. **A** green and red channel. **B** red and blue channel. **C** green and blue channel. The encircled area denotes the SNMP1 positive support cells and the asterisks show the SNMP1-positive OSNs. Scale bar = 10 µm.

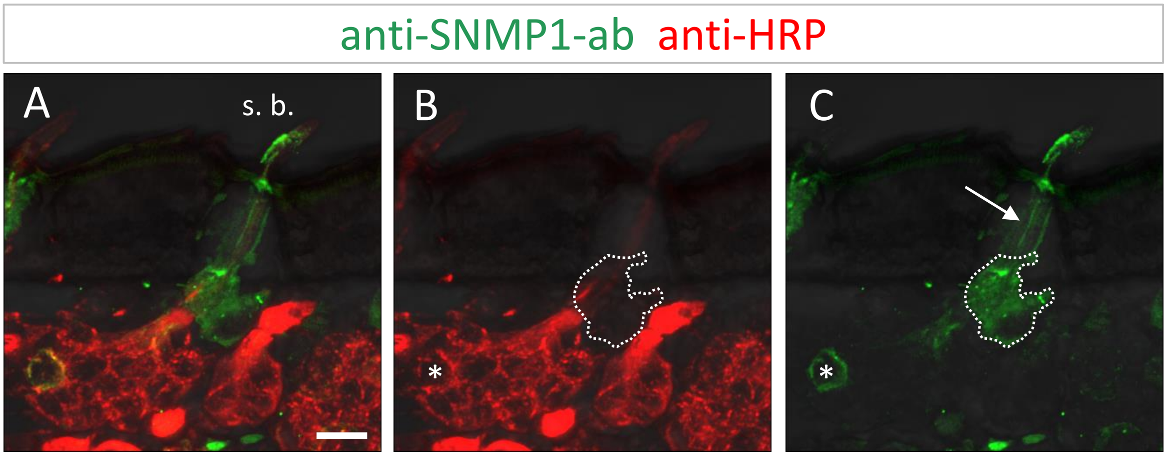

Figure S4. SNMP1 is expressed in distinct OSNs and support cells of a basiconic sensillum in *S. gregaria*. **A-C** FIHC experiment, showing anti-SNMP1-ab immunoreactive cells in the green **(A, C)** and OSNs labelled with anti-HRP in the red **(A, B)** fluorescence channels; the transmitted light channel was overlaid to better display the basiconic sensillum (s. b.). The region encircled denotes the SNMP1-positive support cells; the asterisk marks a SNMP1-expressing OSN within a basiconic OSN cluster. The arrow indicates dendritic labelling by anti-SNMP1-ab. Scale bar = 10 µm.

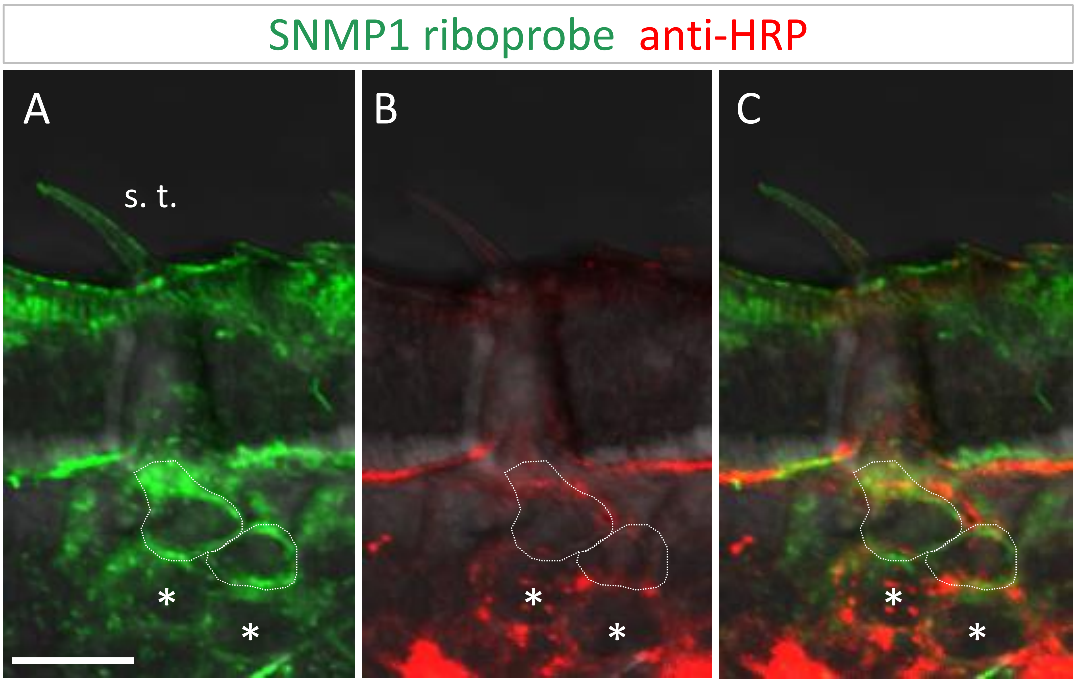

Figure S5. SNMP1 is expressed in support cells and OSNs of a *S. gregaria* trichoid sensillum. The SNMP1-positive cells were visualized by Combined FISH and FIHC using an specific antisense-SNMP1 riboprobe and anti-HRP for labelling of OSN. **A** Cells comprising SNMP1 transcripts visualized in the green fluorescent channel. **B** Labelling of OSNs (red fluorescent channel). **C** merged image of the red and green channels. The encircled areas cells indicated the location of non-neuronal SNMP1-positive support cells while the asterisks show SNMP1-positive OSNs. In all images, the fluorescent channels were overlaid with the transmitted light channels. Scale bar = 10 µm.

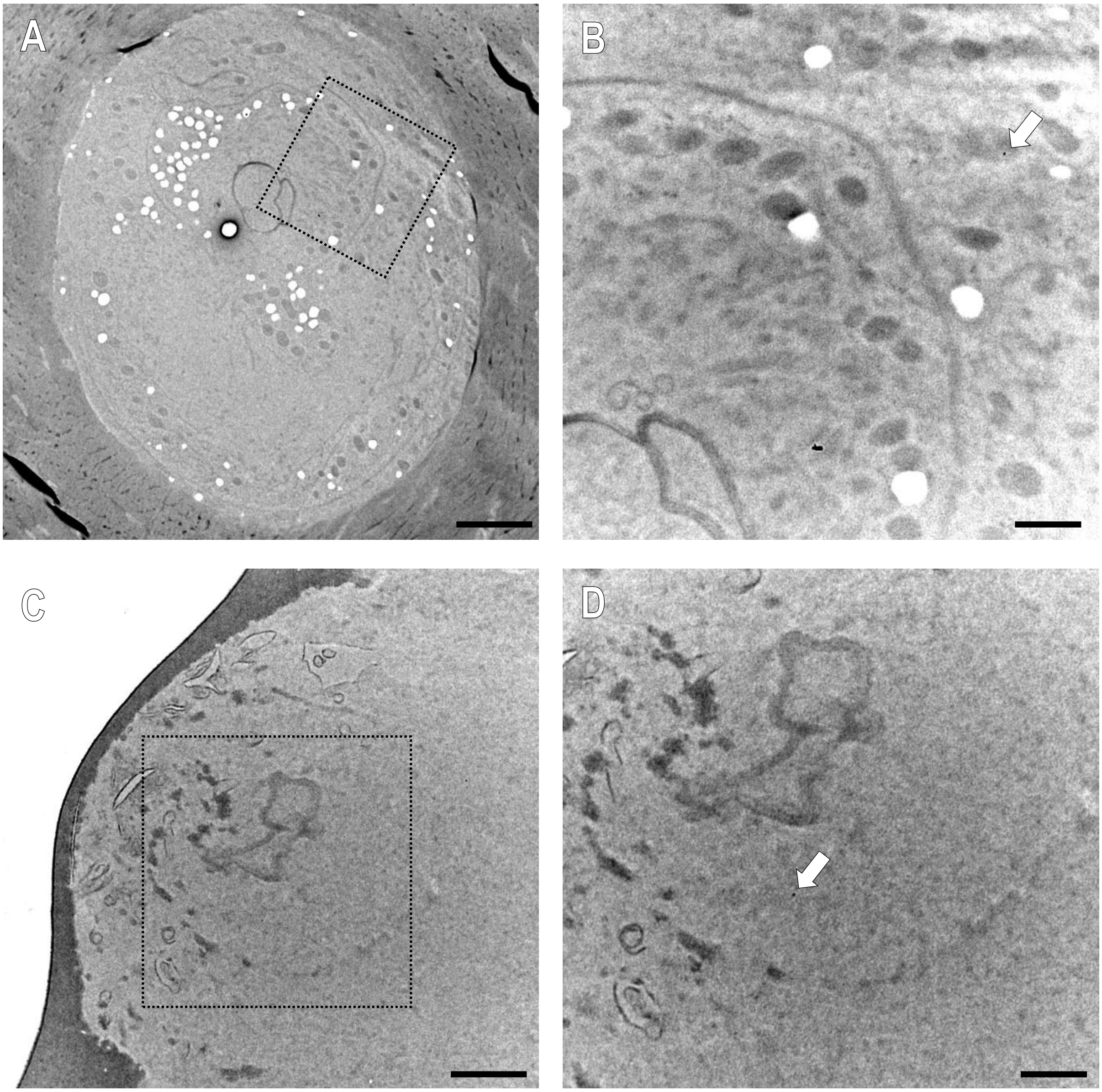

Figure S6. Assessment of secondary antibody binding to ultrathin sections of the *S. gregaria* antenna. Immunogold labelling experiments were conducted omitting the primary antibody, but applying the secondary antibody coupled with colloidal gold. **A** and **C** Overviews of cross section through different sensilla. **B** and **D** show the region boxed in **A** and **C,** respectively, at higher magnifications. Arrows denote single gold grains indicating negligible background labelling of the secondary antibody. Scale bars: A = 2 µm; C = 1 µm; B and D = 500 nm.

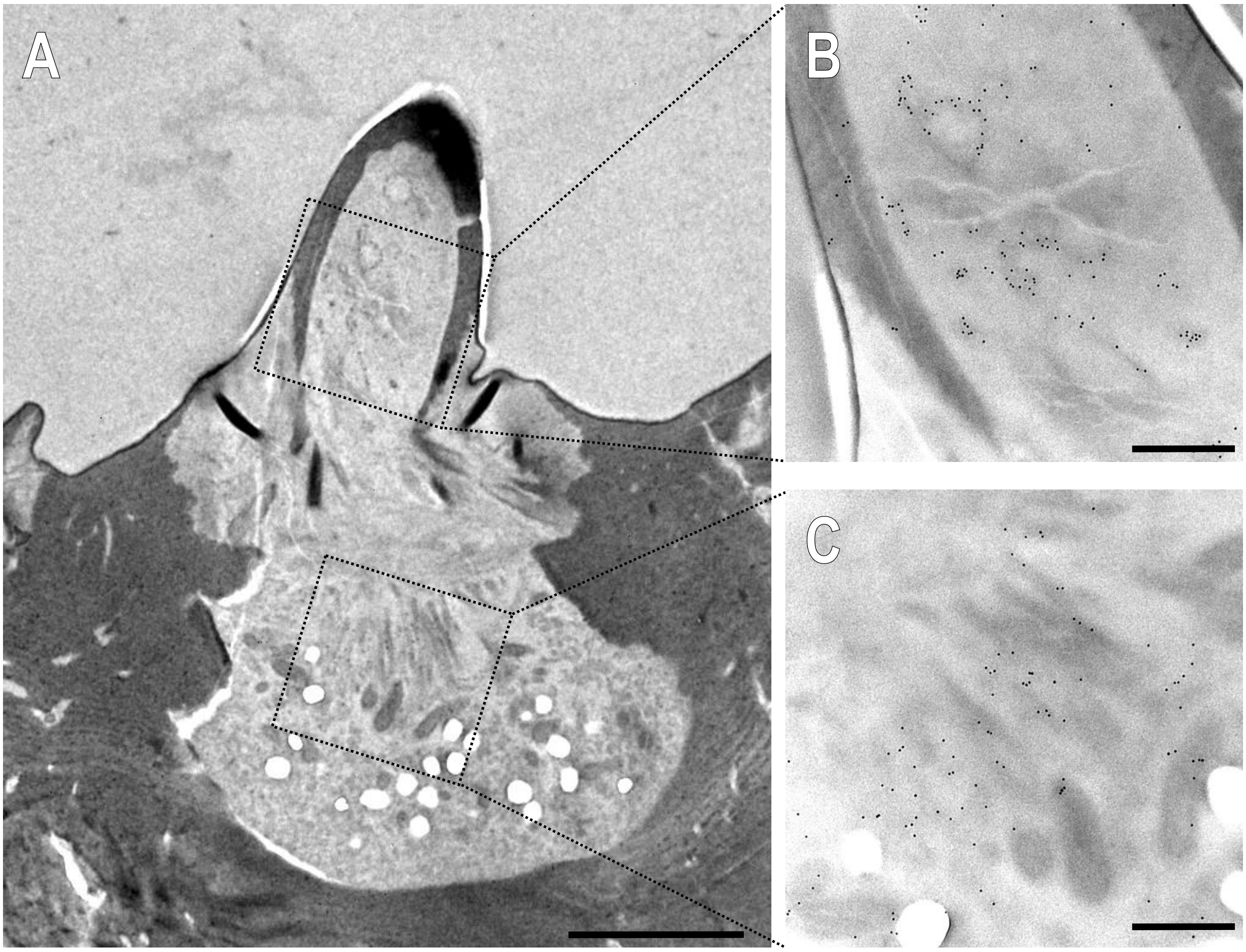

Fig. S7 Localization of SNMP1 within dendrites of OSNs an microvilli structures of support cells in a basiconic sensillum of *S. gregaria*. Ultrathin section of antenna were used in immunogold-labelling experiments with anti-SNMP1-ab and assessed by transmission electron microscopy. **A** Overview image showing a basiconic sensillum. **B** Higher magnification of the sensillum shaft**.** Strong anti-SNMP1-ab immunoreactivity is associated with membranes of dendritic structures within the sensillum shaft. **C** Higher magnifications of an area at the base of the sensillum demonstrating anti-SNMP1-ab labeling associated with microvilli structures of support cells. Scale bars: A = 2 µm; B and C = 500 nm.
